# Remifentanil combined with dexmedetomidine on the analgesic effect of breast cancer patients undergoing modified radical mastectomy and the influence of perioperative T lymphocyte subsets

**DOI:** 10.3389/fsurg.2022.1016690

**Published:** 2022-11-08

**Authors:** Yanjun Zhang, Wei Jiang, Xi Luo

**Affiliations:** ^1^ Department of Breast Surgery, Yongchuan Hospital of Chongqing Medical University, Chongqing, China; ^2^ Department of Anesthesiology, Yongchuan Hospital of Chongqing Medical University, Chongqing, China

**Keywords:** breast cancer, radical mastectomy, dexmedetomidine, remifentanil, perioperative T lymphocyte subsets

## Abstract

**Objective:**

To study the analgesic effect of breast cancer patients undergoing modified radical mastectomy (MRM) and the influence of perioperative T lymphocyte subsets by remifentanil combined with dexmedetomidine.

**Methods:**

80 breast patients were divided into control group and research group based on the anesthesia protocol. Patients in control group was given remifentanil for anesthesia induction and maintenance, and patients in research group was given remifentanil and dexmedetomidine for anesthesia induction and maintenance. We compared the anesthesia time, operation time, surgical blood loss, postoperative wake-up time, extubation time, incidence of adverse reactions, VAS score and T lymphocyte subsets in peripheral blood in the two groups of patients.

**Results:**

The baseline data including age, height, weight and BMI, ASA classification, stage of breast cancer, frequency of neoadjuvant therapy, and surgical characteristics including anesthesia time, operation time and bleeding volume all have no significant difference between two groups (*P* > 0.05). Compared to control group, the time of wake up and extubation in patients of research group were all significantly decreased (*P* < 0.05), and significantly decreased MBP and HR after loading dose of dexmedetomidine in research group (*P* < 0.05). The VAS scores of patients at 4, 8, 12, 16, 20 and 24 h after surgery in the research group are all significantly lower than those in the control group (*P* < 0.05). Before induction of anesthesia, there was no significant difference in the ratio of CD4+, CD8+ and CD4+/CD8+ T lymphocytes in peripheral blood between the two groups (*P* > 0.05). At 1 h during operation and 24 h after operation, the ratio of CD4+ and CD4+/CD8+ cells in the research group was significantly higher than these of the control group (*P* < 0.05), while the ratio of CD8+ cells was lower than that of the control group (*P* < 0.05).

**Conclusion:**

For breast cancer patients undergoing MRM, the use of remifentanil combined with dexmedetomidine can enhance postoperative analgesia and reduce postoperative immunosuppression.

## Introduction

Modified radical mastectomy (MRM) is a common surgical treatment for breast cancer patients. However, immunosuppression caused by surgical anesthesia is the main cause of postoperative infection, immune escape of cancer cells, and metastasis of residual tumor cells (1–3). Anesthesia methods affect neuroendocrine stress responses and immunosuppression, affecting the body's anti-tumor and anti-inflammatory responses, which in turn are associated with cancer recurrence. Therefore, the development of better anesthesia programs is of great significance to improve the postoperative survival rate of breast cancer patients and reduce recurrence. Dexmedetomidine is an effective α2-adrenergic receptor agonist, which not only has the effect of inhibiting the high activity of the central nervous system, but also has the function of antidepressant, anxiolytic and analgesic (4). Importantly, dexmedetomidine is a commonly used adjuvant drug during surgical anesthesia (5). At the same time, dexmedetomidine as an anesthesia adjuvant drug used in MRM not only helps patients to regain consciousness after surgery, but also significantly reduces the incidence of intraoperative and postoperative complications (6, 7).

T lymphocytes are derived from bone marrow pluripotent stem cells and differentiate and mature in the thymus, and they are important immune cells in the human body, and play functions such as cellular immunity and immune regulation in the human body (8, 9). Anesthetic drugs can inhibit the excessive stress response of patients during the perioperative period, directly or indirectly affect the immune function of the body, and affect the specific immune response mediated by T lymphocytes. Previous studies have found that dexmedetomidine combined with remifentanil can achieve better anesthesia effect in patients with breast cancer after MRM, and can significantly reduce the incidence of postoperative adverse reactions in patients with breast cancer (10, 11). However, there are few studies on the effect of dexmedetomidine combined with remifentanil on peripheral blood T lymphocyte subtypes in breast cancer patients under anesthesia in patients with MRM. In this study, we designed to compare the changes of peripheral blood T lymphocyte subtypes in patients undergoing MRM for breast cancer under remifentanil anesthesia alone and remifentanil combined with dexmedetomidine anesthesia. Based on this, the purpose of this study was to study the efficacy of dexmedetomidine in improving the immunosuppressive effect of anesthesia-induced breast cancer patients undergoing MRM.

## Materials and methods

### Ethics statement and patients

This study was approved by the Institutional Medical Ethics Committee of our hospital, and was in accordance with the Declaration of Helsinki. All patients being included in the present study were informed about the content of this study and signed an informed consent form.

A total of 80 breast cancer patients who underwent radical surgery in our hospital from 2019 to 2021 were collected, all patients are female, they were divided into control group (*n* = 40) and research group (*n* = 40) according to the anesthesia protocol. The patients in the control group were 45–64 years old, American Society of Anesthesiologists (ASA) stage I and II are 24 case and 16 case, respectively, breast cancer stages I, II, and III are 15 case, 15 case, and 10 case, respectively. The patients in the research group were 43–64 years old, 25 case and 15 case were ASA stage I and II, respectively; and 16 case, 16 case, and 8 case were breast cancer stages I, II, and III, respectively. Inclusion criteria: (1) diagnosed with breast cancer; (2) younger than 65 years old; (3) ASA stage I-II; (4) undergoing MRM; (5) weight 45–75 kg, height 145–175 cm. Exclusion criteria: (1) Combined with other malignant tumors; (2) Combined with diseases of other tissues and organs such as heart, brain, liver, and kidney; (3) Patients with clinical stage IV breast cancer; (4) Past history of infectious diseases and drug addiction; (4) Cognitive dysfunction or other mental illness.

### Anesthesia protocol

All patients were fasted before operation. After the establishment of anesthesia intravenous channel, the control group was given intravenous injection of 2–4 µg/kg remifentanil (Yichang Renfu Pharmaceutical Co., Ltd.) for induction of anesthesia, and 0.5–2 µg/kg/min remifentanil was used for anesthesia maintenance; 15 min before induction of anesthesia, patients in the research group were intravenously injected with 1 µg/kg dexmedetomidine (Jiangsu Hengrui Medicine Co., Ltd.), and then the anesthesia method was the same as control group. The other anesthetics were used in the same way in the two groups.

### Data collection

According to patient electronic medical records, we collected baseline data including age, height, weight and body mass index (BMI), ASA classification, stage of breast cancer, frequency of neoadjuvant therapy. At the same, we recorded the surgical characteristics including anesthesia time, operation time and bleeding volume, and recorded the anesthesia effect related indicators including mean blood pressure (MBP) and heart rates (HR) at baseline (T0), time after loading dose (T1), induction (T2), intubation (T3), and 30 min after intubation (T4), 60 min after intubation (T5), and 90 min after intubation (T6), and 24 h after surgery (T7). Moreover, the occurrence of adverse reactions in all patients within 48 h after surgery was recorded, including nausea, vomiting, bradycardia, respiratory depression and itching.

### Primary outcome

#### Pain assessment

We used visual analogue scale (VAS) to assess the pain of patients at 4, 8, 12, 16, 20 and 24 h after surgery. The VAS scale is the pain rating scale, which uses a visual analog method to judge the severity of pain. The scoring scale was divided into 10 equal parts using a ruler, with 0 being no pain, 1–3 being mild pain, 4–6 being moderate pain, and 7–10 being severe pain (12, 13).

#### Peripheral blood T lymphocytes

Before induction of anesthesia (preoperative), 1 h during operation (intraoperative) and 24 h after operation (postoperative), we collected 5 ml of peripheral blood from patients, centrifuged the peripheral blood mononuclear cells of patients by density gradient centrifugation, and then added CD3-PE antibody, CD4-TITC antibody and CD8-APC antibody respectively and incubated for half an hour in the dark. Finally, T lymphocyte subtypes were analyzed by flow cytometry.

### Statistical analysis

SPSS 20.0 (SPSS Inc., Chicago, USA) was used to analyze the data in this study. The chi-square test was used to compare the difference of count data between the two groups, and the Student’s *t*-test was used to compare the difference of the measurement data between the two groups. *P* < 0.05 indicates that the difference is statistically significant.

## Results

### Demographic and surgical characteristics

**Figure 1:**
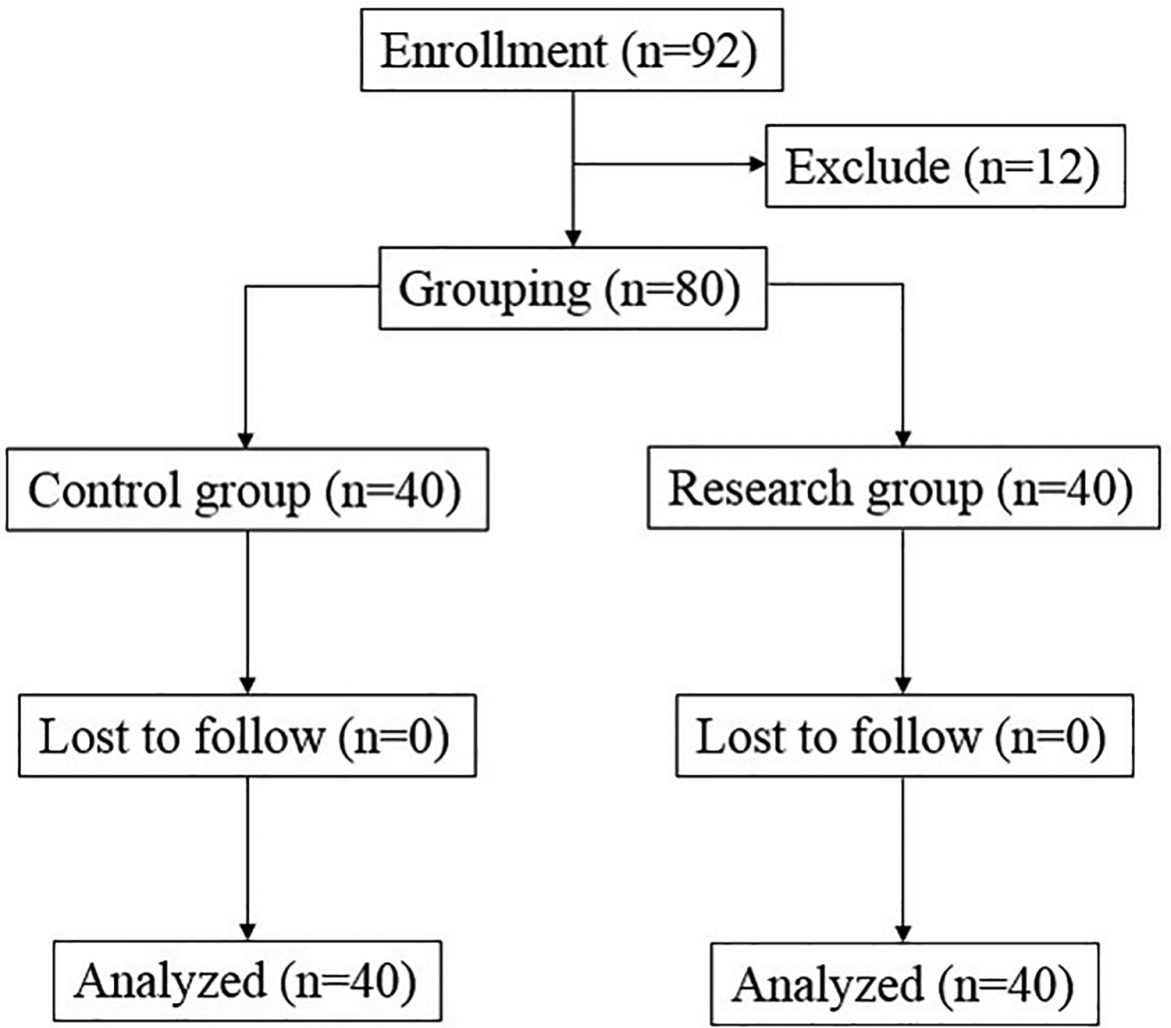
Flow diagram of the study.

There were 92 breast cancer patients being valuated for eligibility, and 12 patients dropped out during the study, and finally 80 patients were involved in the clinical observation experiment. According to the anesthesia protocol, those 80 breast cancer patients were divided into two group: Control group (*n* = 40) and Research group (*n* = 40) (Figure 1). The baseline data including age, height, weight and BMI, ASA classification, stage of breast cancer, frequency of neoadjuvant therapy, and surgical characteristics including anesthesia time, operation time and bleeding volume all have no significant difference between two groups (Table 1).

**Table 1:** Comparison of demographic and surgical indicators between two group (mean ± SD, %).

Groups/variables	Control group (*n* = 40)	Research group (*n* = 40)	*t*/*χ*^2^	*P*
Age (year)	57.6 ± 6.7	57.2 ± 5.1	0.300	0.765
Weight (kg)	60.0 ± 5.9	59.9 ± 5.4	0.059	0.953
Height (cm)	160.0 ± 5.1	160.0 ± 5.0	0.163	0.811
BMI (kg/m^2^)	23.4 ± 1.8	23.4 ± 1.7	0.043	0.966
ASA classification			0.053	0.818
Class I	24 (60.0)	25 (62.5)		
Class II	16 (40.0)	15 (37.5)		
Stage of breast cancer			0.287	0.866
Phase I	15 (37.5)	16 (40.0)		
Phase II	15 (37.5)	16 (40.0)		
Phase III	10 (25.0)	8 (20.0)		
Frequency of neoadjuvant therapy	1.68 ± 0.31	1.70 ± 0.33	0.279	0.781
Anesthesia time (min)	105.7 ± 7.2	105.9 ± 8.0	0.161	0.872
Operation time (min)	90.9 ± 5.4	88.2 ± 5.5	1.154	0.252
Bleeding volume (ml)	62.7 ± 5.3	61.6 ± 5.4	0.879	0.382

### Anesthesia effect related indicators

**Figure 2:**
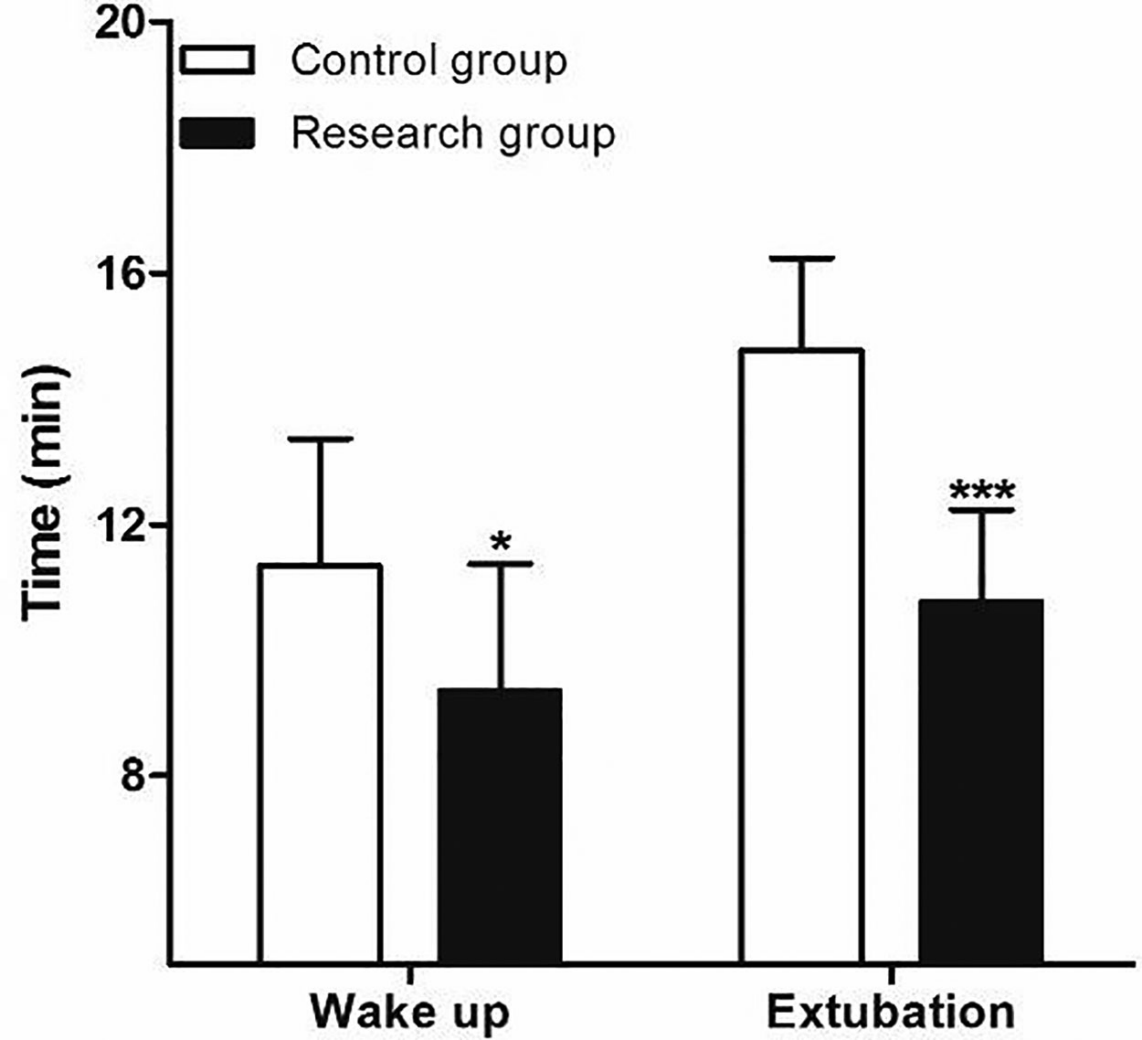
Comparison of postoperative the time of waken up and extubation between two group. Compared with Control group, *P < 0.05 and ***P < 0.001.

**Figure 3:**
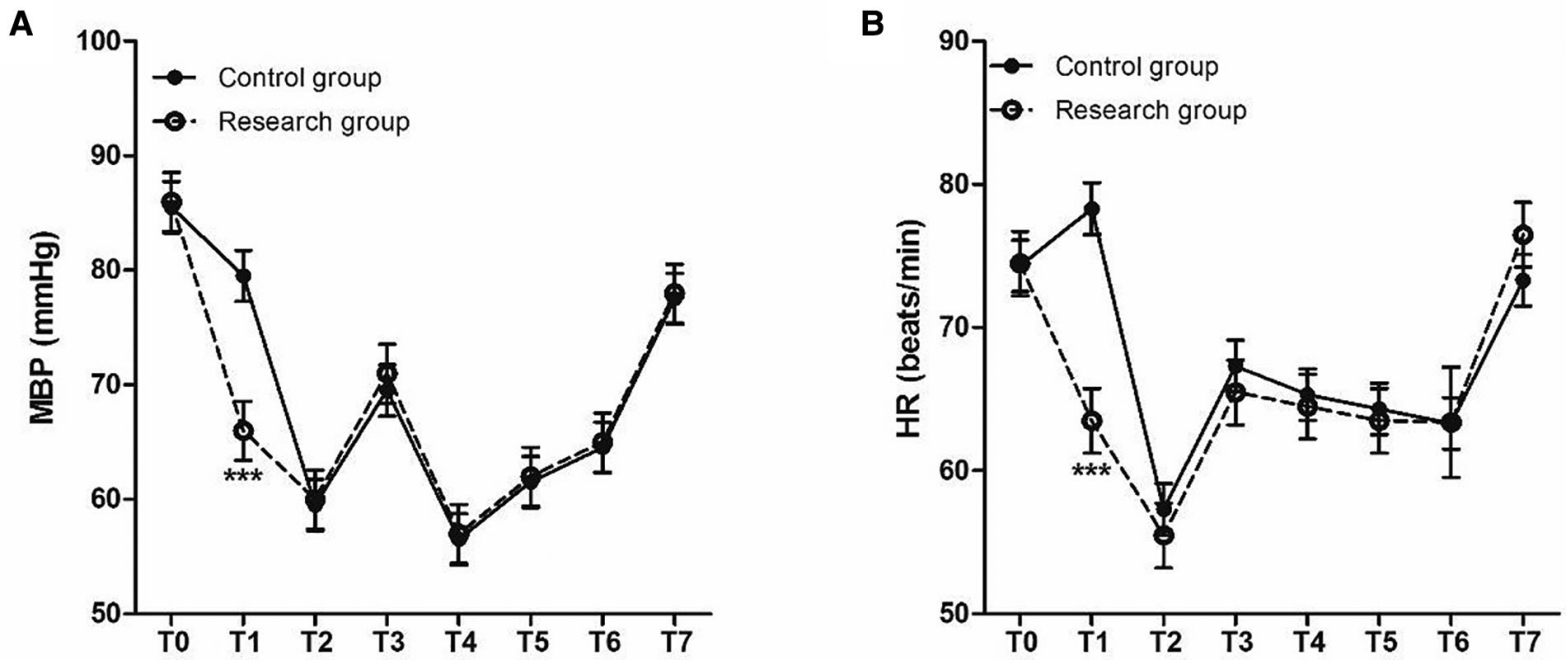
Perioperative HR and MBP at different time points. (**A**) MBP (mmHg) and (**B**) HR (beats/min). T0, baseline; T1, time after loading dose; T2, induction; T3, intubation; T4–T6, 30, 60, and 90 min after intubation; T7, 24 h after surgery.

The time of wake up and extubation in patients of research group were all significantly lower than those in patients of control group (Figure 2). At the same time, the two groups were also comparable with respect to their baseline MBP (Figure 3A) and heart rates HR (Figure 3B) before surgery. Compared to control group, decreased MBP and HR after loading dose of dexmedetomidine in research group (Figures 3A,B).

### VAS score for the analgesic effect

**Figure 4:**
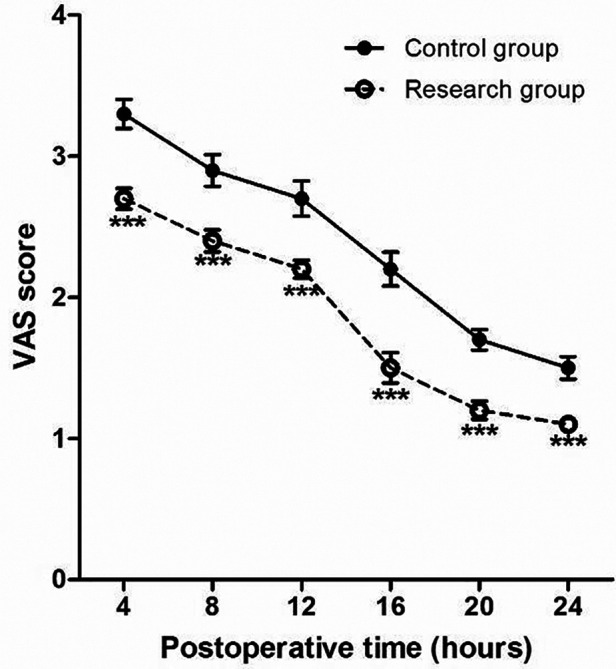
Perioperative VAS score at different time points. Compared with Control group, *P < 0.05 and ***P < 0.001.

The VAS scores of patients at 4, 8, 12, 16, 20 and 24 h after surgery in the research group are all significantly lower than those in the control group (*P* < 0.001) (Figure 4).

### Adverse reactions

Within 48 h after surgery, nausea, vomiting, bradycardia, respiratory depression and itching occurred in 5 case, 4 case, 3 case, 1 case and 1 case in the control group, respectively. However, nausea, vomiting, bradycardia, respiratory depression and itching occurred in 4 case, 3 case, 2 case, 0 case and 2 case in the research group, respectively. There is no significant difference between the control group and research group in the adverse reactions at 48 h after surgery (35.0% vs. 27.5%, *P* > 0.05) (Table 2).

**Table 2:** Comparison of adverse reactions at 48 h after surgery between two groups (*n*, %).

Variables	Control group (*n* = 40)	Research group (*n* = 40)	χ^2^	*P*
Nausea	5 (12.5)	4 (10.0)	0.125	0.723
Vomiting	4 (10.0)	3 (7.5)	0.157	0.692
Bradycardia	3 (7.5)	2 (5.0)	0.213	0.644
Respiratory depression	1 (2.5)	0 (0.0)	1.013	0.314
Itching	1 (2.5)	2 (5.0)	0.346	0.556
Total	11 (27.5)	14 (35.0)	0.524	0.469

### Peripheral blood T lymphocyte subsets

**Figure 5:**
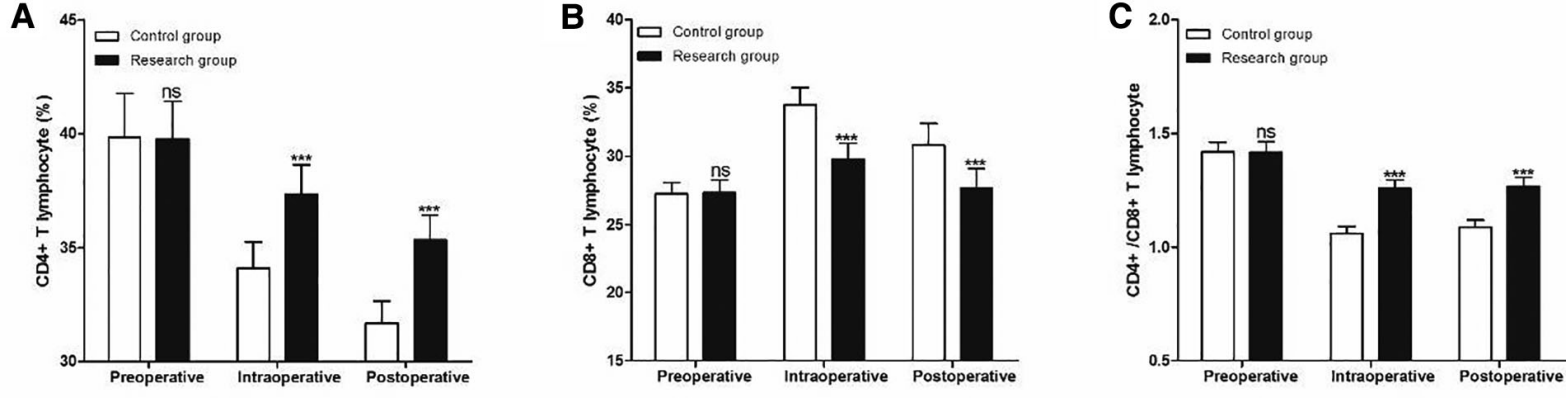
Perioperative peripheral blood T lymphocyte subsets at different timepoints. (**A**) CD4 T lymphocyte, (**B**) CD8 T lymphocyte and (**C**) CD4+/CD8+ T lymphocyte. Compared with Control group, ns *P* > 0.05 and ***P < 0.001.

The two groups were also comparable with respect to their baseline CD4+ (Figure 5A), CD8 + T (Figure 5B) and CD4+/CD8+ (Figure 5C) T lymphocyte (*P* > 0.05). At 1 h during the operation (intraoperative) and 24 h after the operation (postoperative), the CD4+ and CD4+/CD8+ T cells in the research group were significantly higher than those in the control group (*P* < 0.001), while the CD8+ T cells were significantly lower than those in the control group(*P* < 0.001) (Figure 5).

## Discussion

Breast cancer is one of the most common malignancies, with approximately 2.2 million new cases of breast cancer and more than 680,000 deaths worldwide each year (14). In China, there are 420,000 new cases of breast cancer and 120,000 deaths from breast cancer each year (15, 16). There are five main treatments for breast cancer, including surgery, radiotherapy, chemotherapy, targeted therapy and immunotherapy. Among them, preoperative neoadjuvant chemoradiotherapy combined with surgery has become a classic treatment strategy for patients with advanced breast cancer (17, 18). However, immunosuppression caused by surgical anesthesia in breast cancer patients undergoing MRM will not only increase the risk of postoperative infection, but also increase the risk of tumor cell immune escape and increase the probability of postoperative recurrence (19, 20). Therefore, a scientific and appropriate anesthesia scheme is of great significance to the survival and postoperative recovery of patients after radical resection of adenocarcinoma.

Dexmedetomidine is a highly selective α2 adrenergic receptor agonist, which has the advantages of rapid onset of action, short duration of action, sedative and analgesic effects, and no respiratory depression, and is a widely used anesthesia adjuvant drug (9, 10). In this study, we found that the anesthesia time, operation time, and blood loss during the operation did not increase in the research group with additional dexmedetomidine for anesthesia, indicating that dexmedetomidine does not affect the surgical process of patients undergoing MRM for breast cancer, which is consistent with the results in the study by Das et al. (21). Das et al. found that the addition of dexmedetomidine can not only significantly reduce the consumption of anesthetics in patients undergoing MRM for breast cancer, but also significantly shorten the postoperative breathing satisfaction time, eye opening time and extubation time. In addition, both this study and the study of Das et al. found that the incidence of postoperative adverse reactions in patients with dexmedetomidine anesthesia was lower, indicating that the addition of dexmedetomidine for anesthesia can not only improve the anesthesia effect, but also is safe and effective.

In addition, in this study, we also used the VAS scale to evaluate the pain status of patients at different times after surgery, and the results found that the VAS scores of the patients in the research group were significantly lower than those in the control group within 24 h after surgery, it shows that the postoperative analgesia effect of the patients in the research group was better, which is basically consistent with the research results of Liu et al. (22). A meta-analysis of 12 clinical studies reported that dexmedetomidine was a favorable anesthetic adjuvant in breast cancer surgery, which can relieve postoperative pain (22). However, the results of the study by Yang et al. was different from this study, and they found that the VAS scores of breast cancer patients with dexmedetomidine added at 2, 8 and 24 h after surgery were not significantly different from those of control patients (23). Therefore, the effect of dexmedetomidine on postoperative pain in patients with MRM for breast cancer may be related to the time of pain assessment, the included population, and the subject of the assessment.

T lymphocytes are a key component of the human immune system. By making appropriate activation responses to relevant antigens, they lead and coordinate various immune responses of the immune system to ensure that the body effectively removes invading pathogens or diseased cells, and avoids immune diseases occur on its own (24, 25). CD is short for leukocyte differentiation antigen, CD3 is a class of antigens on the surface of T lymphocytes, and CD3+ refers to mature T lymphocytes. CD3 binds to the T cell receptor and can transmit antigen signals to T lymphocytes (26, 27). CD4+ lymphocytes, namely helper T cells, have the function of assisting humoral and cellular immunity (28, 29). CD8+ lymphocytes, namely cytotoxic T cells, have the function of killing target cells and are activated by direct binding of MHCI to antigens (30, 31). In this study, we found that the ratio of CD4+ and CD4+/CD8+ cells in the research group was significantly higher than these of the control group, while the ratio of CD8+ cells was lower than that of the control group at 1 h during operation and 24 h after operation, indicating dexmedetomidine can significantly reduce anesthesia-induced immunosuppression in patients undergoing MRM for breast cancer.

## Conclusion

In conclusion, adding dexmedetomidine to anesthetize patients with MRM for breast cancer can not only enhance the anesthesia effect of remifentanil, but also reduce postoperative pain and the incidence of postoperative adverse reactions, and reduce postoperative immunosuppression by regulating T lymphocyte subsets.

## Data Availability

The original contributions presented in the study are included in the article/Supplementary Material, further inquiries can be directed to the corresponding author/s.
